# Qualitative and semi-quantitative ultrasound assessment in delta and Omicron Covid-19 patients: data from high volume reference center

**DOI:** 10.1186/s13027-023-00515-w

**Published:** 2023-05-27

**Authors:** Vincenza Granata, Roberta Fusco, Alberta Villanacci, Francesca Grassi, Roberta Grassi, Federica Di Stefano, Ada Petrone, Nicoletta Fusco, Stefania Ianniello

**Affiliations:** 1 Division of Radiology, “Istituto Nazionale Tumori IRCCS Fondazione Pascale – IRCCS di Napoli”, 80131 Naples, Italy; 2 Medical Oncology Division, Igea SpA, Naples, Italy; 3 Department of Radiology and Diagnostic Imaging, National Institute for Infectious Diseases IRCCS Lazzaro Spallanzani, 00149 Rome, Italy; 4 Division of Radiology, “Università degli Studi della Campania Luigi Vanvitelli”, Naples, Italy; 5 Italian Society of Medical and Interventional Radiology (SIRM), SIRM Foundation, Via della Signora 2, 20122 Milan, Italy

**Keywords:** Ultrasound assessment, Covid-19, Delta variant, Omicron variant

## Abstract

Objective: to evaluate the efficacy of US, both qualitatively and semi-quantitatively, in the selection of treatment for the Covid-19 patient, using patient triage as the gold standard. Methods: Patients admitted to the Covid-19 clinic to be treated with monoclonal antibodies (mAb) or retroviral treatment and undergoing lung ultrasound (US) were selected from the radiological data set between December 2021 and May 2022 according to the following inclusion criteria: patients with proven Omicron variant and Delta Covid-19 infection; patients with known Covid-19 vaccination with at least two doses. Lung US (LUS) was performed by experienced radiologists. The presence, location, and distribution of abnormalities, such as B-lines, thickening or ruptures of the pleural line, consolidations, and air bronchograms, were evaluated. The anomalous findings in each scan were classified according to the LUS scoring system. Nonparametric statistical tests were performed. Results: The LUS score median value in the patients with Omicron variant was 1.5 (1–20) while the LUS score median value in the patients with Delta variant was 7 (3–24). A difference statistically significant was observed for LUS score values among the patients with Delta variant between the two US examinations (*p* value = 0.045 at Kruskal Wallis test). There was a difference in median LUS score values between hospitalized and non-hospitalized patients for both the Omicron and Delta groups (*p* value = 0.02 on the Kruskal Wallis test). For Delta patients groups the sensitivity, specificity, positive and negative predictive values, considering a value of 14 for LUS score for the hospitalization, were of 85.29%, 44.44%, 85.29% and 76.74% respectively. Conclusions: LUS is an interesting diagnostic tool in the context of Covid-19, it could allow to identify the typical pattern of diffuse interstitial pulmonary syndrome and could guide the correct management of patients.

## Introduction

Although almost three years have passed since the first documented case of Covid-19 infection, and despite the introduction of vaccines that are effective in preventing severe forms of the disease, the management of the infected patient remains a health emergency, especially about hospital admission [1-34]. Globally, at May 2023, there have been 766,404.339 confirmed cases of COVID-19, including 6,935.958 deaths, reported to WHO [35]. As of 26 October 2022, a total of 12,830.378.906 vaccine doses have been administered [35].

While the imaging methods have improved the diagnosis, also thanks to the contribution of artificial intelligence techniques, thanks to which it is also possible to define a stratification of the patient's risk, both in terms of disease progression and death, and in terms of post-infection sequelae [36-54], the progress in the field of treatment still remains to be understood. To date, a defined treatment for Covid-19 infection has not been introduced. There may be different therapeutic options, many of them based on symptom control, and the possibility of using one class or another depends on the disease severity and timing of onset [55-66]. Monoclonal antibodies (mAbs) have transformed the treatment of several diseases, including cancer and inflammatory or autoimmune conditions, and are a new first line for the treatment of infectious diseases. An unprecedented number of mAbs have been developed over the past year to combat coronavirus disease 2019 (COVID-19) [67-73]. However, considering that RNA viruses, as SARS-CoV-2, are evolving biological entities, it is known that these entities can escape the immunity elicited by infection, vaccination, or mAb administration [74]. Antiviral agents represent a major advance in the COVID-19 patient therapeutic management, leading to a substantial reduction in SARS-CoV-2-related complications and mortality [73]. Currently available agents are expensive and the efficacy has not been demonstrated for several subtypes [73]. In this scenario, a correct patient selection should be carried out according to a rigorous study protocol that considers the time of drug administration so as the imaging tools that could objectively select the patient [75-91]. Although ultrasounds (US) have been suggested by various scientific societies as a tool to be used in selected cases [92-94], the possibility of a wide using make them a suitable tool in the patient risk assessment [95-104]. The introduction of different evaluation scores also make the assessment objectively and non-operator dependent [105-115].

Aim is this study is to assess the US role and efficacy, as qualitatively as semi-quantitatively diagnostic tool, in the Covid-19 patient treatment selection, using patient triage as gold standard.

## Methods

### Patient characteristics

This retrospective study was conducted according to the guidelines of the Declaration of Helsinki and approved by the Institutional Ethics Committee IRCCS L. Spallanzani. Data acquisition and analysis were performed in compliance with the protocols approved by the National Institute for Infectious Diseases IRCCS L. Spallanzani Ethics Committee (Ethical Approval Number 164, 26 June 2020). The Local Ethical Committee board, considering the ongoing epidemic emergency, renounced patient informed consent.

Out-patients admitted to the Covid-19 clinic to be treated with mAb or retroviral therapies, within five days of Covid-19 symptoms onset, and undergoing lung ultrasound were selected between December 2021 and May 2022, according to the following inclusion criteria: (1) patients with proven variant COVID-19 infection Omicron and Delta; (2) patients with known COVID-19 vaccination with at least two doses; (3) patients with ultrasound images at baseline. Exclusion criteria were: (1) patients without ultrasound images at baseline, (2) patients with no clinical follow-up.

### Ultrasound procedures and images assessment

Lung US (LUS) was performed by expert radiologists, with > 5 years of experience in lung evaluation, and an adequate personal protective equipment. An US system (Acuson Juniper, Siemens-Healthineers, Germany) with convex 3.5–5 MHz and linear 4–8 MHz probes, dedicated to COVID-19 patients, was used. Covers for the probes and the US machine console were used during the procedure.

Examinations were performed with patients in a sitting position, systematically scanning the anterior and posterior portions of each hemithorax. A convex probe was initially used to obtain a panoramic view of the pleural line and ultrasound artifacts associated with the status of the lung parenchyma (A-lines, B-lines, and consolidations). Then, to obtain a more detailed study of the appearance of the pleural line and subpleural abnormalities, the radiologist used a linear probe.

According to Mongodi et al. [116], LUS score was computed in six regions per hemithorax: regions 1 and 2, anterior regions; regions 3 and 4, lateral regions; and regions 5 and 6, posterior regions. Complete examinations taken 10–15 min for each patient.

Images were saved using a dedicated software and reviewed by radiologist after the assessment, to avoid prolonged exposure with the infected patient.

The presence, location, and distribution of abnormalities, such as B-lines, thickening or ruptures of the pleural line, consolidations, and air bronchograms, were evaluated.

The anomalous findings in each scan were also classified according to the scoring system (LUS score) proposed by Soldati et al. [117] (0 = regular pleural line, “A” lines present; 1 = notched pleural line, focal B lines; 2 = broken pleural line, subpleural consolidations; and 3 = blank lung with or without consolidations).

### Patient clinical data

Patient Clinical data, including vital signs and oxygen saturation in room air at the moment of admission, SARS-CoV-2 testing, timing of symptom onset, and main comorbidities, were also retrieved from each clinical record.

### Statistical analysis

Continuous data were expressed in terms of median values and range. Chi square test and Kruskal Wallis test were used to verify differences among groups. Kolmogorov–Smirnov was used methods to test the normality of the data.

*P* value < 0.05 was considered significant for all tests.

The statistical analyses were performed using the Statistics and Machine Toolbox of MATLAB R2021b (MathWorks, Natick, MA, USA).

## Results

Our study group included thirty-six patients (median age 58 years, range 28–82 years) with Omicron variant subjected to US evaluation and forty-three patients (median age 68 years, range 31–84 years) with Delta variant with US evaluation. No Omicron patients were re-assessed by US, since this evaluation it is not required according to symptoms. Among Delta patients, 16 patients were subjected to US examination in the follow-up (8 days after the baseline US as median value; range 6–23 days after the baseline US).

Kolmogorov–Smirnov verified the normality of the data (*p* value < 0.05).

**Fig. 1:**
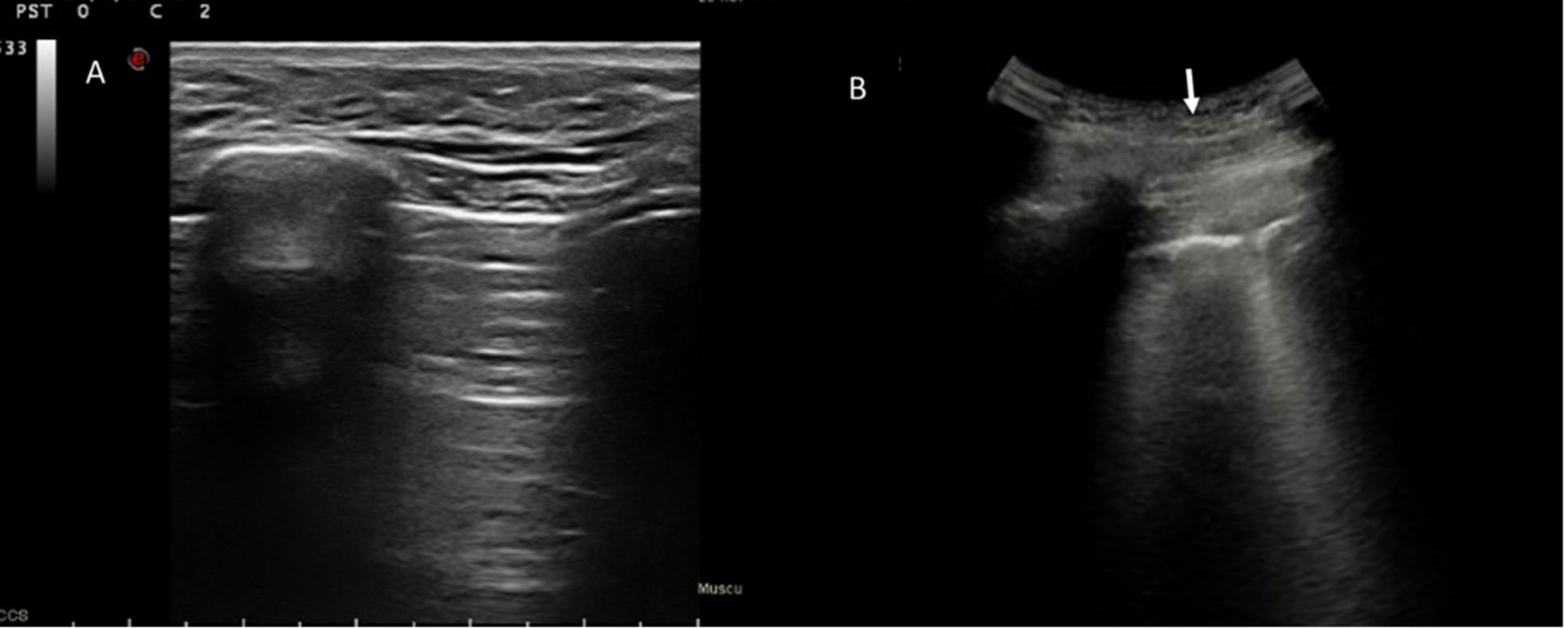
Omicron patient. In **A** R2 zone, normal findings with line A. In **B** L5, single B lines with Lung Score of 3 (arrow)

**Fig. 2:**
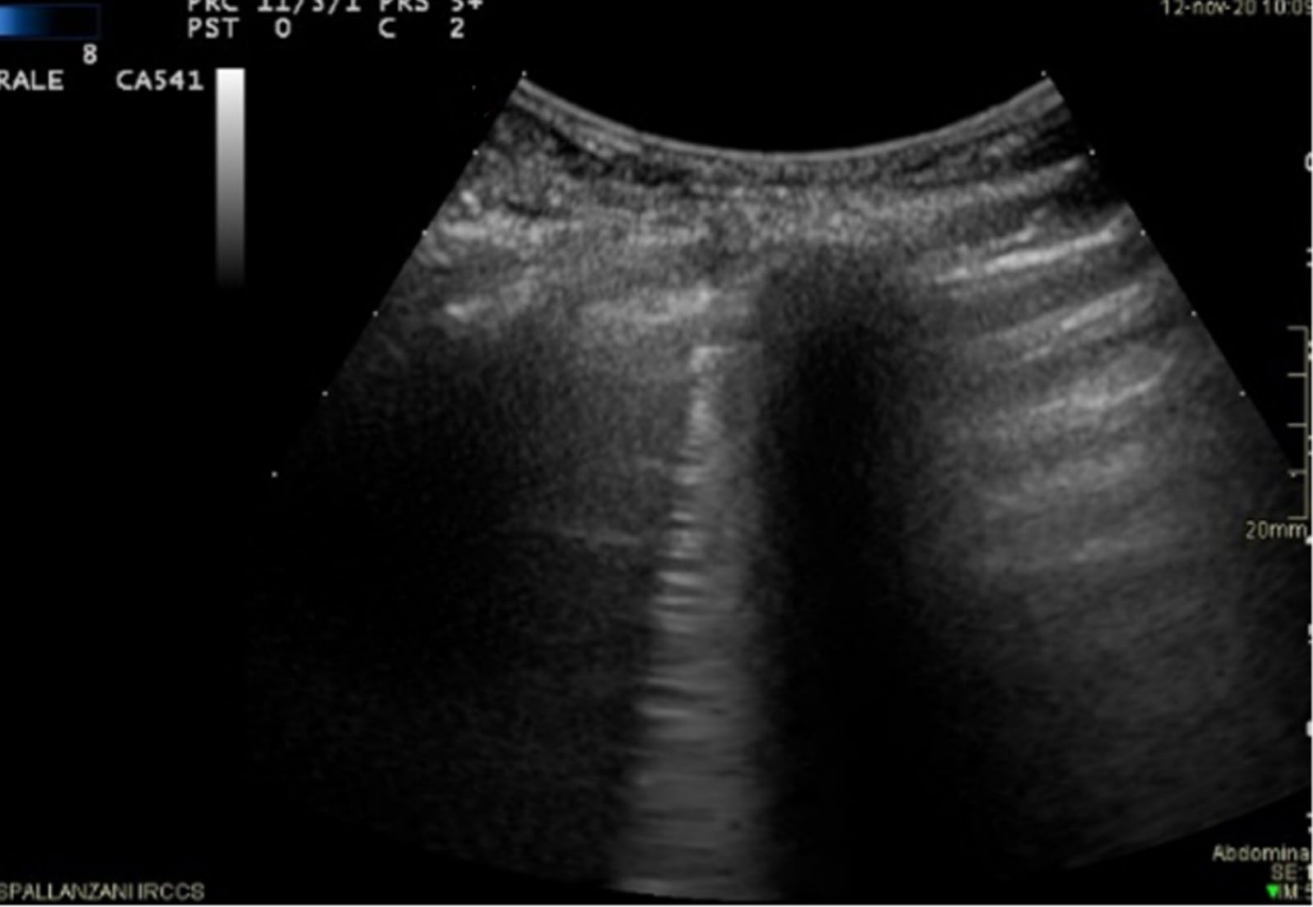
Omicron patient with single B line in L1 (arrow); Lung Score of 3

**Fig. 3:**
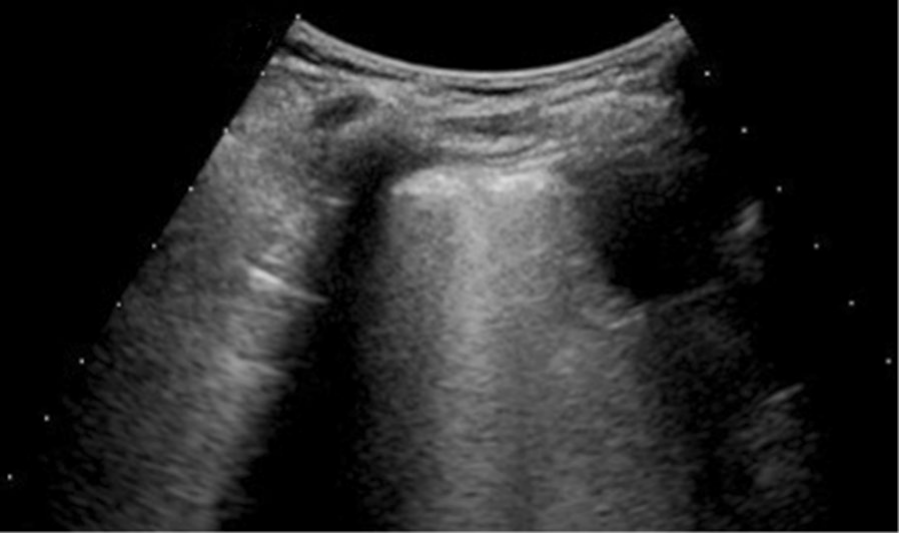
Omicron patient with confluent B lines (arrow) with high grade pleural line thickening in L2; Lung Score of 20

**Fig. 4:**
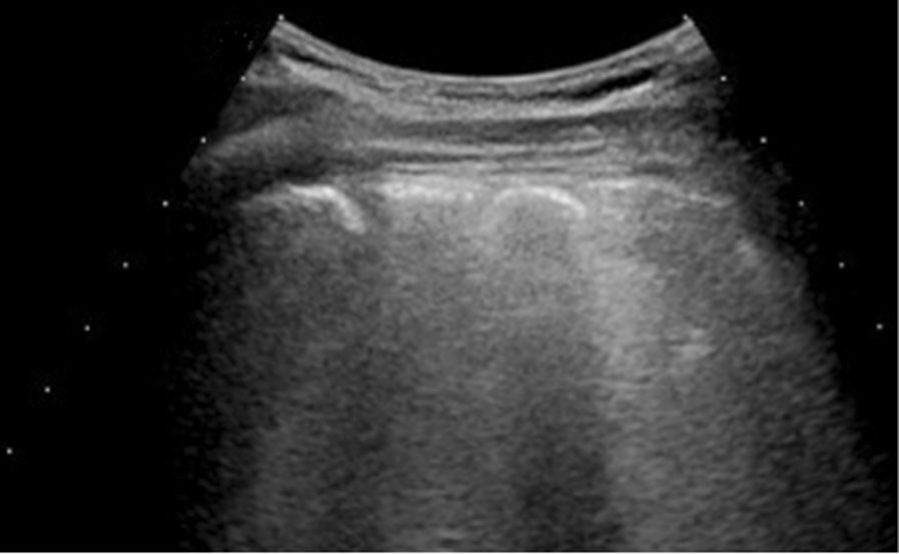
Delta patient with confluent B lines (arrow) with medium grade pleural line thickening in R2; Lung Score of 14

With regard to qualitative assessment, among Omicron patients: 5 had single B-lines (Figs. 1, 2), 4 confluent B-lines, 7 low grade pleural line thickening, 2 medium grade pleural line thickening, 1 high grade pleural line thickening and one consolidative pattern (Fig. 3).

**Fig. 5:**
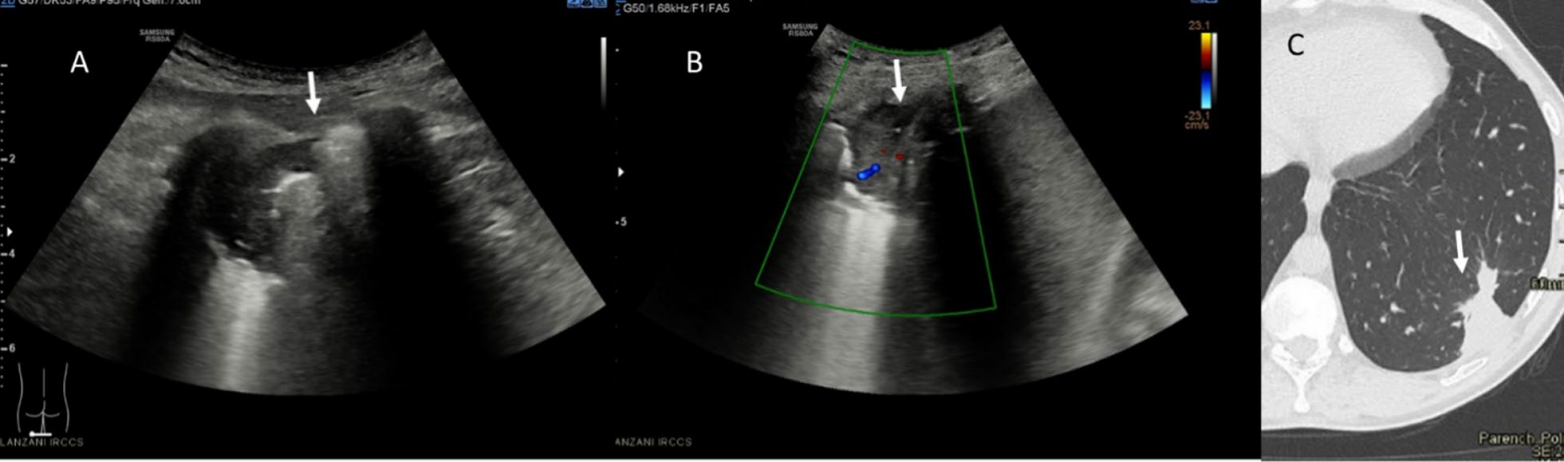
Delta patient: in **A** (US assessment) and **B** (Color-Doppler assessment) consolidative pattern (arrows) in L6; Lung Score 24. On CT assessment (**C**), arrow shows consolidative pattern

Among Delta patients: 3 had single B-lines, 12 confluent B-lines, 6 low grade pleural line thickening, 6 medium grade pleural line thickening (Fig. 4), 5 high grade pleural line thickening and 3 consolidative pattern (Fig. 5).

Among Omicron patients, 25 patients had fever (range 37.5°–40°), 22 cough, 22 pharyngitis, 1 dyspnea, 2 fibromyalgia and 2 had headache.

Among Delta patients: 4 dyspnea, 22 fever (range 37.5–40), 22 cough, 11 fibromyalgia, 10 headache and 2 dyspepsia.

Table 1 reports LUS and US patterns score median value and range for single pattern. There was a statistically significant difference in median LUS score values between different patient subgroups grouped by US pattern for both the Omicron and Delta groups (*p* value ≪ 0.01 at Kruskal Wallis test).

**Table 1:** LUS score value for enrolled patient

	Omicron No. of patients	Delta No. of patients	LUS score median value (range) Omicron	LUS score median value (range) Delta
Single B-lines	5	3	1 (1–2)	5 (3–7)
Confluent B-lines	4	12	8 (3–20)	12 (5–18)
Low grade pleural line thickening	7	6	2 (2–5)	4.5 (3–7)
Medium grade pleural line thickening	2	6	3.5 (3–4)	9 (7–13)
High grade pleural line thickening	1	5	8	16 (8–23)
Consolidative pattern	1	3	13	13 (7–24)
Negative	16	8	0	0

There was no statistically significant difference in the US pattern between the Omicron and Delta patient groups (*p* value = 0.43 at Chi square test).

No statistical differences were found between Lung score and symptoms (*p* value = 0.25 at Kruskal Wallis test) and between US pattern and symptoms (*p* value = 0.37 at Kruskal Wallis test).

**Fig. 6:**
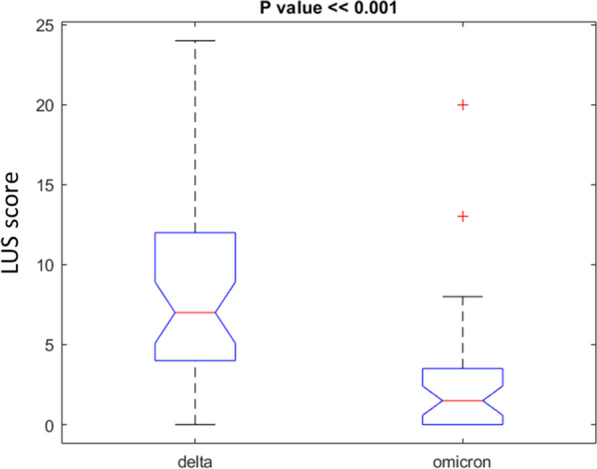
Boxplot of LUS score values among the 36 patients with Omicron variant compared to the 43 patients with Delta variant

A difference statistically significant was observed for LUS score median values among the patients with Omicron variant compared to the patients with Delta variant (*p* value < 0.001 at Kruskal Wallis test, Fig. 6). The LUS score median value in the patients with Omicron variant was 1.5 (1–20) while the LUS score median value in the patients with Delta variant was 7 (3–24).

**Fig. 7:**
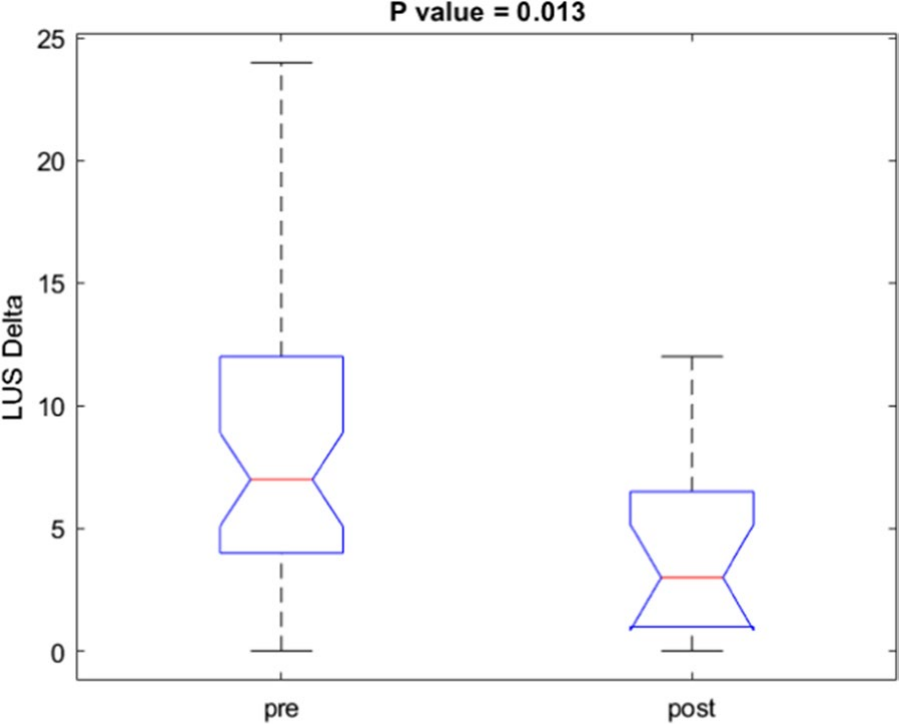
Boxplot of LUS score delta values among the 16 patients with Delta variant between the two US examinations

A difference statistically significant was observed for LUS score values among the patients with Delta variant between the two US examinations (*p* value = 0.045 at Kruskal Wallis test, Fig. 7).

The LUS score median value in the patients with Delta variant in the follow-up was 3.

**Fig. 8:**
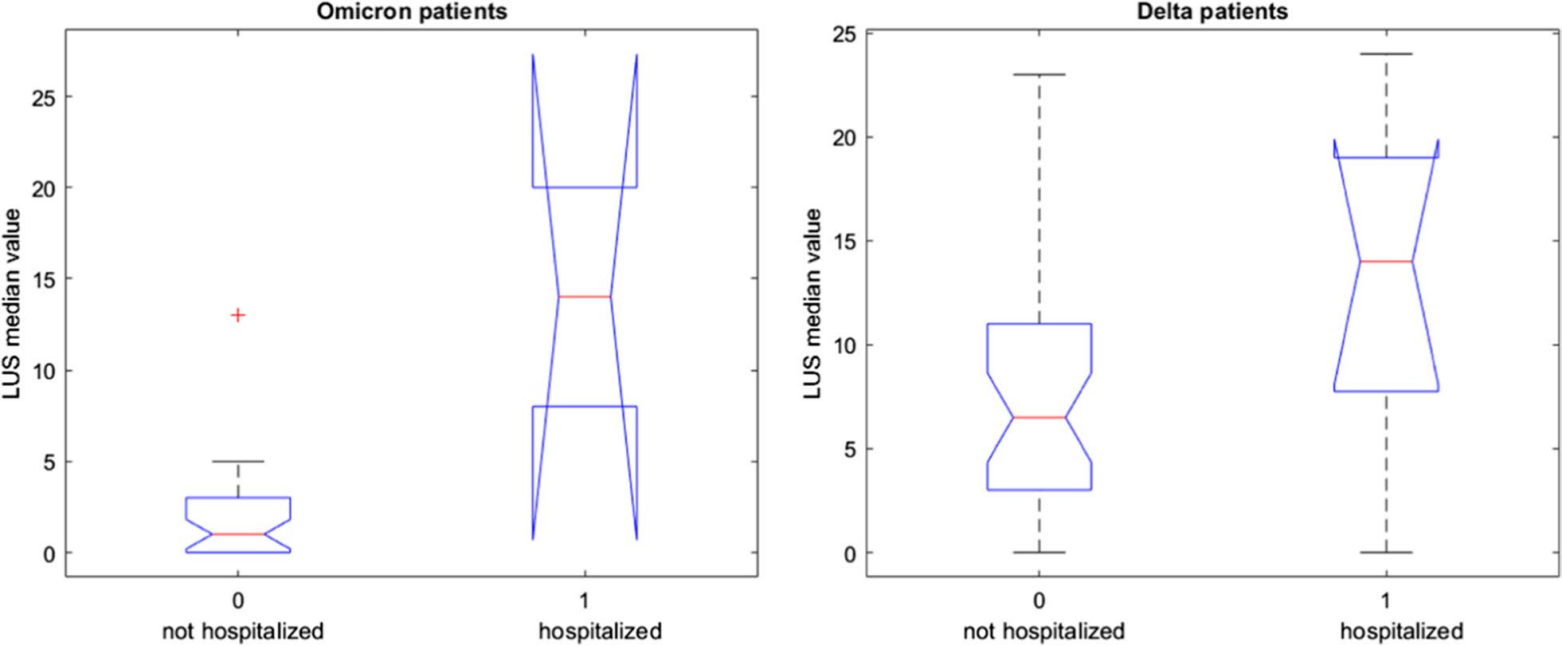
Boxplots of LUS score median values between the hospitalized and not hospitalized patients in Omicron and Delta patient groups (9 patients were hospitalized while among the Omicron patients 2 patients were hospitalized)

Among the Delta patients, 9 patients were hospitalized while among the Omicron patients 2 patients were hospitalized. There was a statistically significant difference in median LUS score values between hospitalized and non-hospitalized patients for both the Omicron and Delta groups (*p* value = 0.02 on the Kruskal Wallis test). For both Delta and Omicron patients, the cut-off value of LUS score for the hospitalized patients was 14 (Fig. 8).

For Delta patients groups the sensitivity, specificity, positive and negative predictive values, considering a value of 14 for LUS score for the hospitalization, were of 85.29%, 44.44%, 85.29% and 76.74% respectively. Considering that only 2 patients were hospitalized in Omicron patient groups the performance of LUS score was not calculated.

## Discussion

Covid-19 infection remains a health emergency considering the high transmission rate of the virus, the variability of the clinical symptoms and the need for careful monitoring of patients, since, although 80% of patients have mild symptoms, approximately 14% of patients have moderate to severe disease with 5% becoming critically ill [35]. Also, considering that a proper coronavirus treatment has not yet been established and several therapies are aimed at resolving the symptoms, an assessment of the risk of an infected patient, both in terms of disease progression and the need for admission to intensive care unit, is crucial [118, 119]. In this context, the need for an objective evaluation of pulmonary involvement appears evident.

Today, several Computed Tomography (CT) severity scores have been proposed [120-125] and, although CT is thought to play a key role COVID-19 patient diagnostic work-up [4-13, 108, 126-143], however the transport of infected patients for a CT scan followed requires necessary decontamination procedures that makes this procedure risky and time consuming [118]. According to the WHO [35], COVID‐19 patient, that require hospitalization for moderate–severe disease, will need supplementary oxygen and consistent observing to facilitate early recognition and escalation of the deteriorating patient. In this scenario, LUS is an effective diagnostic tool, since this procedure do not require patient transport since it can be performed in patient room [118].

In COVID-19 pneumonia, LUS helps to identify the typical pattern of diffuse interstitial pulmonary syndrome, characterized by multiple or confluent B-lines, pleural line thickening, pleural line irregularity, and consolidations [127]. LUS findings correlate with CT findings and have several advantages such as lack of radiation exposure, repeatability during follow-up, low cost, and easier application [127, 128].

We evaluated 36 Omicron patients and 43 Delta patients, which have been assessed with US, showing that no statistical differences were found between Lung score and symptoms and between US pattern and symptoms. Considering this result, it is clear as the symptoms should not be the only data to be evaluated in order to hospitalize a patient, while the possibility of objectifying the disease allows an optimization of patient management, avoiding overtreatment and under treatment.

With regard to severity, although, there was no statistically significant difference in the US pattern between the Omicron and Delta patient groups, a difference statistically significant was observed for LUS score values among the patients with Omicron variant compared to the patients with Delta variant. In fact, the LUS score median value in the patients with Omicron was 1.5, while in Delta patients was 7. These results are in line with previous studies that demonstrated that in-hospital mortality was 7.6% for alpha, 12.2% for delta, and 7.1% for omicron [129]. Among unvaccinated patients with hospitalized Covid-19, severity on the WHO Clinical Progression Scale was higher for delta than alpha and lower for omicron than delta [129]. Compared with unvaccinated patients, severity was lower for vaccinated patients for each variant, including alpha and omicron [129].

Among Delta patients, 9 patients were hospitalized while among Omicron patients 2 patients were hospitalized. There was a statistically significant difference in median LUS score values between hospitalized and non-hospitalized patients for both the Omicron and Delta groups. For both Delta and Omicron patients, the cut-off value of LUS score for the hospitalized patients was 14. For Delta patient groups the sensitivity, specificity, positive and negative predictive values, considering a value of 14 for LUS score for the hospitalization, were of 85.29%, 44.44%, 85.29% and 76.74% respectively. However, due to the small number of patients and the extreme overlapping box plots, the evidence for the difference between the two groups should be further validated.

Our results are in line with the data reported by previous study [130]. A recent systematic review showed as LUS can be used at home assistance to prevent unnecessary hospital admission, since LUS, integrated with clinic and physical evaluation, results in more accurate diagnosis of COVID-19. Additionally, LUS can be used in the prognostic stratification of patients with pneumonia through the extension of specific patterns and their evolution to the consolidation phase in emergency setting [130]. These data were, also, confirmed by multicenter prospective cohort study [129]. The authors evaluated 398 patients and reported that LUS predicts mechanical ventilation, ICU admission, and high-flow oxygen treatment but not survival [108].

During follow-up, a difference statistically significant was observed for LUS score values among the patients with Delta variant between the two US examinations. The LUS median score value in the patients with Delta variant in the follow-up was 3. These results are similar to Lichter et al. results [131], which showed that clinical deterioration was associated with increased follow-up LUS scores, mostly due to loss of aeration in anterior lung segments. A multicenter study evaluated patients with COVID-19-related acute respiratory distress syndrome (ARDS) with at least one LUS study within 5 days of initiating invasive mechanical ventilation [132] evaluating 137 patients and demonstrating that the global LUS score was associated with successful exit from mechanical ventilation regardless of ARDS severity, but not with 28-day mortality [132].

LUS is interesting diagnostic tool for assessing the severity of COVID-19 related pneumonia since this examination could potentially decrease or eliminate the need for hospitalization. In addition, decreasing global LUS score was associated with a better treatment response and clinical outcome.

Our study has several limitations: (a) patient selection, considering only patients admitted to the Covid-19 clinic to be treated with mAbs or retroviral treatment, this could cause biased selection; (b) retrospective study; (c) The prognostic value of changes in LUS score over time could not be assessed for all patients. Post-treatment changes in LUS scores should be further evaluated as a monitoring tool for lung parenchymal re-aeration; (d) heart failure or extensive emphysema are not considered in our study, however, they are not comorbidities that may affect the ultrasound image and the presence, location, and distribution of abnormalities, such as B-lines, thickening, or ruptures of the pleural line, consolidations and air bronchograms that were evaluated on the images.

## Conclusion

LUS is attractive diagnostic tool in Covid-19 setting, it could allowing to identify the typical pattern of diffuse interstitial lung syndrome, and could guide the proper patient management. We found a difference in median LUS score values between hospitalized and non-hospitalized patients for both the Omicron and Delta groups. A possible cut-off value of LUS score for the hospitalized patients could be 14.

## Data Availability

All data are reported in the manuscript.
